# Impacts of longitudinal training in patient-centered medicine on trainee physicians’ career choices and practice behaviors

**DOI:** 10.12688/mep.20993.1

**Published:** 2025-09-18

**Authors:** Memoona Hasnain, Jennifer Grage, Kanwal Haque

**Affiliations:** 1 Family & Community Medicine, University of Illinois Chicago College of Medicine, Chicago, Illinois, USA; 2 Pediatrics, Northwestern University Feinberg School of Medicine, Chicago, Illinois, USA

**Keywords:** Primary care, patient-centered care, health workforce development

## Abstract

**Purpose:**

To investigate the impacts of *The Patient-centered Medicine (PCM) Scholars Program* – a longitudinal curriculum designed to inculcate attitudes, values, and competencies to practice patient-centered medicine, with a special focus on underserved populations, addressing social determinants of health and health disparities, and advancing health equity and social justice – on graduates’ career choices and practice behaviors.

**Methods:**

Cross-sectional online survey of 2010–2017 program graduates (N=191) and analysis of residency placement data 2010–2024 from an institutional website. Sixty-three graduates responded to the survey, translating to a 33% response rate.

**Results:**

Eighty-five percent of respondents reported that the program prepared them to provide patient-centered care for underserved and vulnerable patients. The most important PCM skills applied in practice included: building relationships with patients, communicating well with patients, and practicing collaborative decision-making. For residency training, 53% of graduates chose primary care settings, and 67% chose to work in underserved settings. The open-ended data revealed that students strongly valued the following aspects of the PCM Scholars Program: exposure to patient experience, dedication to serving the underserved, and building relationships with patients.

**Conclusions:**

Our study provides evidence regarding the impacts of the PCM Scholars Program graduates’ career choices and their competencies to practice patient-centered care. The PCM Scholars Program has led to meaningful contributions to training the future healthcare workforce, to address the needs of our evolving patient populations with a special focus on advancing social justice and health equity. Long term, this work will continue to focus on bridging the gap between health professions education and practice and creating transformative educational innovations responsive to the need for a highly trained healthcare workforce that can meet the challenges of the 21 ^st^ century.

## Introduction

There is growing recognition that **patient-centered care** - *care that is respectful of and responsive to patients’ preferences, needs, and values -* results in greater patient satisfaction and adherence, improved outcomes, reduction in unnecessary services, greater physician fulfillment, and reduced malpractice claims, all without significant increases in time and money ^1, 2^. Patient-centered care is the cornerstone of the New Model of Care advocated by the Future of Family Medicine Project ^3^, and it is one of six quality domains designated by the Institute of Medicine ^4^. The Institute of Medicine defines patient-centered care as “Healthcare that establishes a partnership among practitioners, patients and their families (when appropriate) to ensure that decisions respect patients’ wants, needs, and preferences”. This approach identified as a key domain of quality care ^4^, emphasizes active patient involvement and shared decision-making throughout the treatment process. Vulnerable populations, such as low-income individuals, uninsured persons, immigrants, racial and ethnic minorities, incarcerated individuals, and older adults, face even greater barriers to patient-centered care. Evidence from leading national agencies, such as the National Institutes of Health, the Institute of Medicine, and the Centers for Disease Control and Prevention, indicates that healthcare disparities continue to exist across diverse populations. Racial and ethnic minorities are less likely to receive routine medical procedures and experience a lower quality of health services, even when age, severity of medical conditions, income, and insurance status are comparable ^5– 10^. These disparities exist at both national and local levels and patient unrest and dissatisfaction are growing.

To respond to the persisting healthcare disparities and to advance health equity, there are numerous calls for redesigning medical education to prepare the health workforce. The recommendations involve addressing the evolving health needs of patients and communities, with a substantial shift of medical school and residency education to community-based settings, focusing on social determinants of health and experiential learning, as well as prioritizing the social accountability mission of academic institutions ^11– 25^. A survey distributed to medical schools by the American Medical Association to investigate the level of commitment to teaching social determinants of health to their students found that integrating social determinants of health education in basic or clinical sciences is not currently prioritized ^26^. In addition, a scoping review of existing patient-centered curricula to understand programs designed to teach primary care residents about social determinants identified gaps in curriculum development, implementation, and evaluation ^27, 28^. The review highlighted the need to create a rigorous evaluation of delivering social determinants curricula that influence residents’ behavior ^27^. These calls are grounded in a common framework to better prepare future physicians for the context and challenges of care in real-life practice settings. There is a paucity, however, of training programs designed to inculcate in medical students and residents the attitudes and competencies necessary to provide such care, and the *quality chasm* in our healthcare system remains a reality.

### Program development history

**Figure 1.**
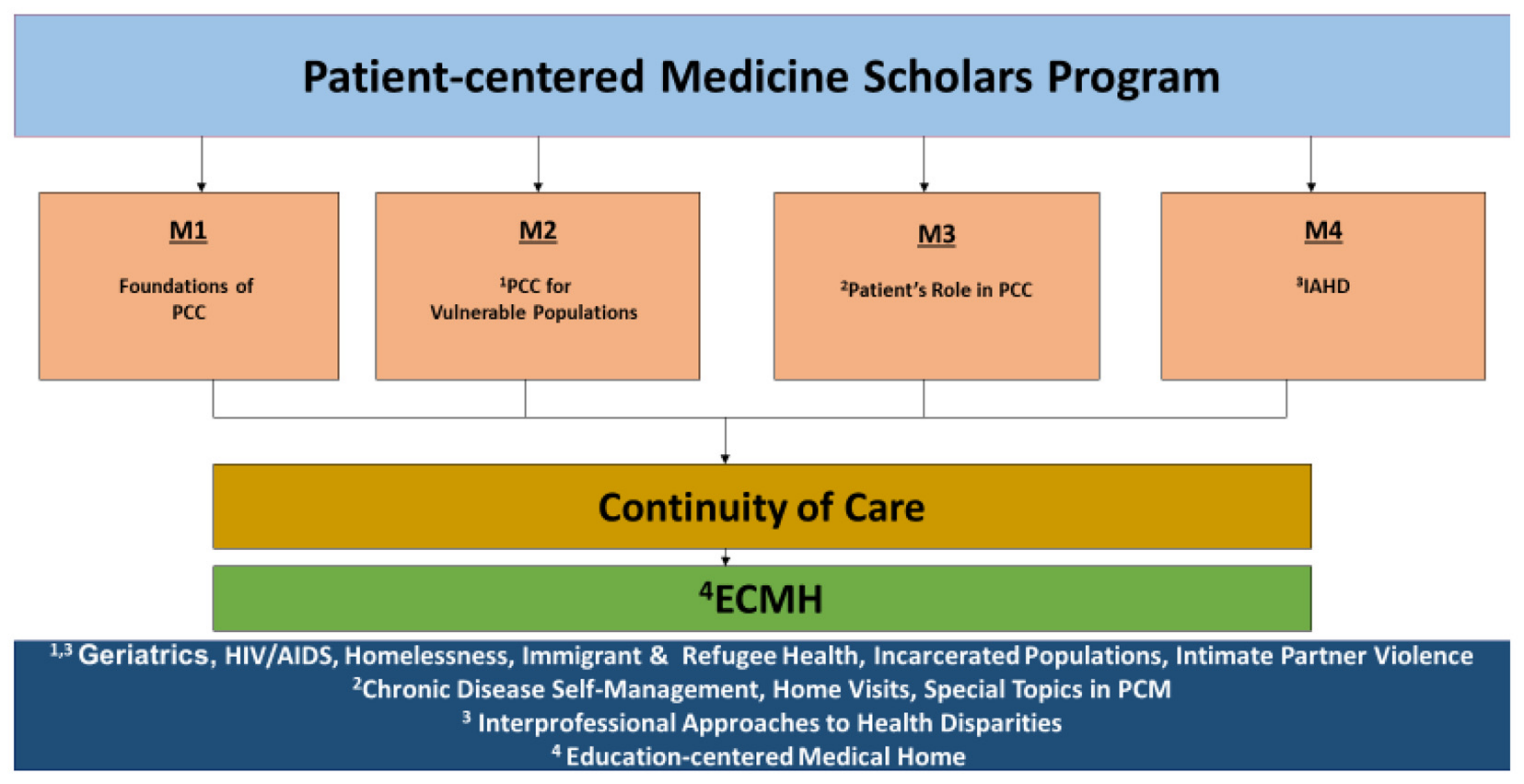
Patient-centered Medicine Scholars Program Components. The PCM Scholars Program is designed to prepare medical students for primary care practice and to serve medical underserved populations. The M1 year is the Foundations of Patient-centered care (PCC); M2 is the PCC for Vulnerable Populations; M3 is the Patient’s Role in PCC; and M4 is the Interprofessional Approaches to Health Disparities (IAHD). In addition, students have the option to medically participate in continuity clinical experiences thought the Education-centered-Medical Home (ECMH), fostering patient-centered care longitudinal relationships with patients. In the M3 year, the contents areas are chronic disease self-management, home visits, and special topics in PCM. Content areas for the M2 and M4 years include, geriatrics, HIV/AIDS, homelessness, immigrant and refugee health, incarcerated populations, and intimate partner violence.

To bridge this gap, in 2002 the University of Illinois Chicago College of Medicine Department of Family and Community Medicine began offering cultural competency workshops for third-year clerkship students and family medicine residents through funding from the Illinois Department of Public Health. In 2005, we piloted a Service Learning Program (SLP) for 2 ^nd^ year medical students. The SLP was developed with small funding from the American Medical Student Association (AMSA). In 2007, with larger funding from the Health Resources and Services Administration (HRSA), we expanded the SLP to a longitudinal continuity of care curriculum, *The Patient-centered Medicine (PCM) Scholars Program*. The first author (MH) is the founding and continuing Director of the Program. The PCM Scholars Program Components are presented in Figure 1.

**Figure 2.**
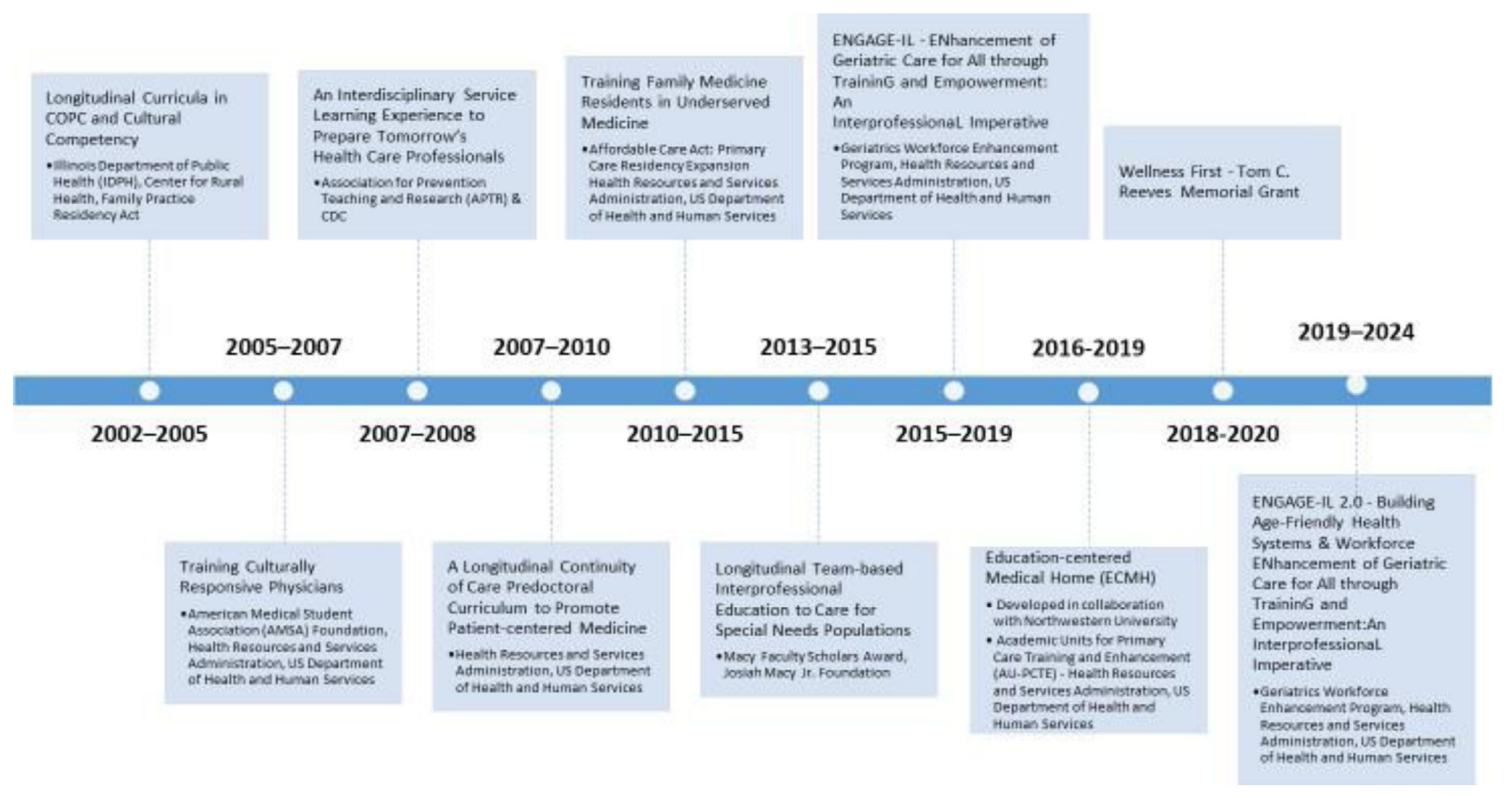
Journey in program development. This timeline illustrates the development and evolution of the PCM Scholars Program. Early initiatives focused on culturally responsive care and longitudinal primary care education began in 2002. Between 2005 and 2015, efforts expanded to include interdisciplinary service learning, continuity of care, and team-based care for underserved populations. From 2015 onward, initiatives such as the ENGAGE-IL programs emphasized interprofessional collaboration, geriatrics, and workforce development. Funding was provided by a range of sources.

Given the importance of interprofessional education, in 2008, we piloted an interprofessional learning experience for medicine and pharmacy students with funding from the Association of Prevention Teaching and Research (APTR). In 2013, the fourth-year component was redesigned as an interprofessional learning experience with funding from the Josiah Macy Junior Foundation ^29^. The Summary of PCM Scholars Program Development is presented in Figure 2.

### Program design

The primary purpose of the PCM Scholars Program is to equip health professions trainees with attitudes, values, and competencies likely to ensure that they can and will practice patient-centered medicine for all patients, with a special focus on underserved/vulnerable populations, addressing social determinants of health and health disparities, and advancing health equity and social justice. Students are selected by applying to the program and have the opportunity to participate throughout their medical school education. The program aims to develop leaders and scholars, focusing on cultivating humanism and empathy by blending the art and science of medicine. The program is built on John Dewey’s “learning by doing” philosophy ^30^ and David A. Kolb’s experiential learning model ^31^.

**Figure 3.**
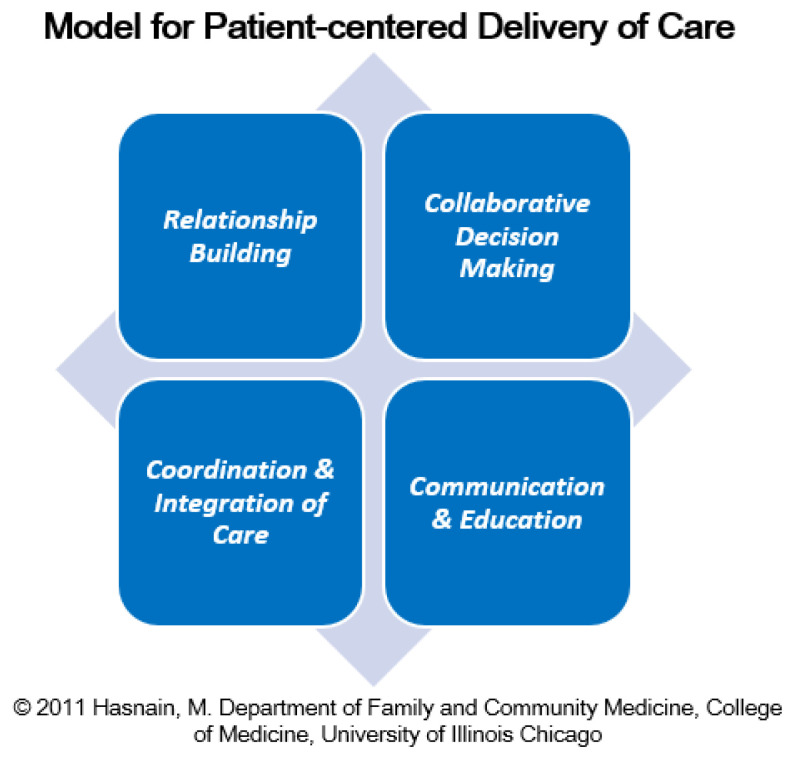
Model for Patient-centered Delivery of Care. The depicted model outlines four core components of patient-centered care: (1) Relationship Building; (2) Collaborative Decision Making; (3) Coordination and Integration of Care and; (4) Communication and Education.

The PCM Scholars Program’s ***conceptual framework,*** “ *Model for Patient-centered Care*,” developed by the first author (MH), is organized as four interlinked core elements: *Relationship Building, Collaborative Decision-Making, Coordination and Integration of Care, and Communication and Education*. The framework is presented in Figure 3.

A central thread of the curriculum is students’ ongoing involvement with patients, including underserved patients, over the course of their training. This “continuity of care” is a core element of learning. Students participate in longitudinal patient care and service-learning activities with diverse patients, including underserved and vulnerable populations, and engage in community-based participatory research to improve healthcare for vulnerable populations. Each program component builds on concepts and skills necessary for practicing patient-centered medicine ^32^. Detailed program syllabi for each component are available upon request.

The program was created on the foundation that patient-centered medicine and continuity of care lead to better patient experiences and outcomes ^1, 33^. Now, in its 20 ^th^ year, the program is fully integrated into the medical school curriculum, and students have reported that it is one of the best training experiences in medical school. Over the years, the program has been refined based on the need to build a curriculum structure that integrates longitudinal integrated primary care and public health education with community-based participatory research (CBPR) and quality improvement (QI) research in an interprofessional learning environment, essential for preparing health care leaders with skills to address the rising burden of key public health concerns effectively.

The PCM Scholars Program was initiated with external funding and sustained after the completion of the funding period (see Figure 2). Program evaluation for measuring learning outcomes utilizes Kirkpatrick’s Four-Level Training Evaluation Model, which assesses program participant reactions, learning, behaviors, and results ^34^. While data had been collected on participants’ reactions and learning outcomes, we had not gathered evidence about how participation in the PCM Scholars Program impacts graduates’ career choices and behaviors related to their patient care practices; the ***purpose*** of this paper is to share findings of a study exploring these aspects for the medical students who participated in the program. A separate paper will share the findings of the interprofessional aspect of the program.

## Methods

The study was reviewed and approved by the UIC Institutional Review Board (IRB) as exempt on June 19, 2017 [IRB Protocol Number: 2007-0567].

### Study design, setting, and participants

The study employed a cross-sectional design. Eligible participants were University of Illinois Chicago, College of Medicine PCM Scholars Program Graduates who participated in one or more curricular components (M1-M4) of the Program from 2010–2017 and had spent at least one year in a post-graduate residency program. Two hundred and twenty-eight graduates met both requirements; 191 were reachable via contact information on file and through internet search. Our program office maintains a database of current PCM scholars and graduates. The residency training site for each graduate was abstracted from the University of Illinois Chicago College of Medicine Match Results website ^35^. The HRSA medically underserved areas (MUAs) designation tool ^36^ was used to assess whether the graduates decided to pursue their residency in a MUA, this tool is publicly available and does not require a copyright license for use. MUAs are defined as a shortage of primary care health services within geographic areas ^37^.

### Survey development and deployment

**Figure 4.**
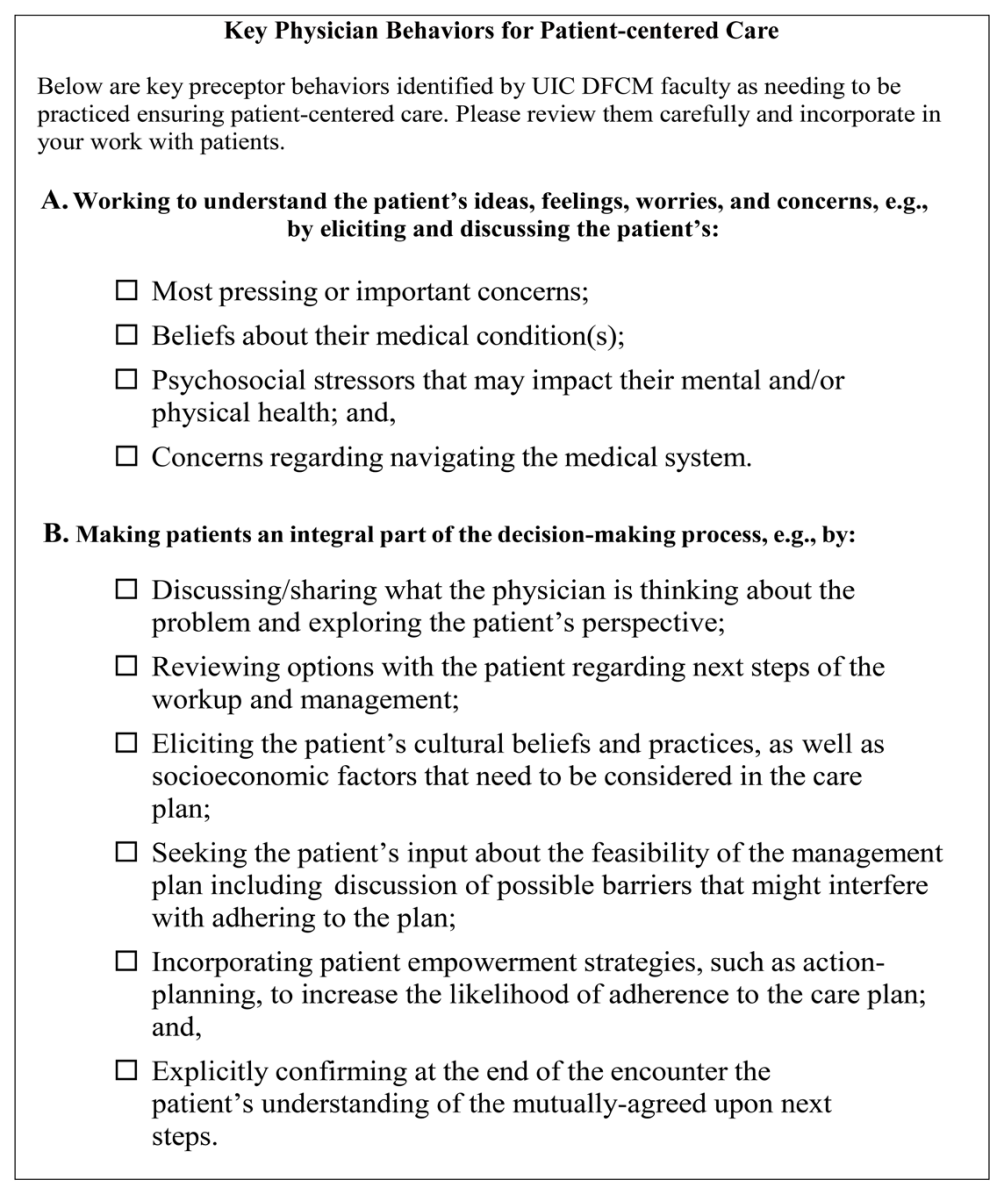
PCM Scholars Program - Key Physician Behaviors for Patient-centered Care. This figure outlines core physician behaviors identified by the first author and the physician faculty in the UIC Department of Family and Community Medicine as essential for practicing patient-centered care. These behaviors are organized into two domains: Understanding the Patient’s Perspective and Shared Decision Making. These behaviors serve as a foundation for cultivating patient-centered practice among medical trainees in the PCM Scholars Program.

The first two authors (MH and JG) developed the UIC COM Graduates PCM Scholars Program Follow-Up Survey. The survey consisted of questions that assessed various elements of patient-centered care as previously defined by the PCM Scholars Program. The *Key Physician Behaviors for Patient-Centered Care* are presented in Figure 4. Questions were also adapted from a previously validated patient-centered care survey created by the first author ( *M4 Learning Outcomes Survey*), and additional questions were created to assess the specific aims of the program. The answer choices for survey items used a five-point Likert rating scale. The survey also included open-ended questions. The survey was piloted with a few students before being distributed to all eligible participants. The final survey was distributed via Qualtrics, a web-based survey platform.

### Data analysis

Statistical Package for the Social Sciences (SPSS) Statistics 24 was utilized to conduct the descriptive analysis of quantitative items. The Likert Scale responses were collapsed, converting the five categories (Almost Always, Usually, About Half the Time, Seldom, and Almost Never) to three (Almost Always, About Half the Time, and Almost Never).

Data from the open-ended response questions included in the survey were analyzed using content analysis ^38^.

## Results

Of the 191 potential participants, 63 graduates participated in the survey, yielding a 33% response rate.

### Quantitative analysis

***Sample demographics.*** The respondents were 44% female, 35% White or Caucasian, 8% Asian, 6% Hispanic or Latino, 3% African American or Black, 2% Middle Eastern or Arab, and 2% Multiracial. The average age was 32 years. The characteristics of the PCM scholars are presented in Table 1.

**Table 1.** Characteristics of PCM Scholars, N = 63.

Characteristics	Frequency (%)
**Age (M = 32.1, SD = 3.1)**	38 (60.3)
**Gender**	
Female	28 (44.4)
Male	11 (17.5)
Other	0 (0.0)
Missing	24 (38.1)
**Racial/Ethnic Identity**	
African American or Black	2 (3.2)
Asian	5 (7.9)
Hispanic or Latino	4 (6.3)
Middle Eastern or Arab	1 (1.6)
Multiracial	1 (1.6)
Native American/Alaskan	0 (0.0)
Other	3 (4.8)
White or Caucasian	22 (34.9)
Missing	25 (39.7)
**PCM Program Influence** **on Choice of Specialty**	
Yes	21 (33.3)
No	23 (36.5)
Missing	19 (30.2)
**PCM Program Influence** **on Choice of Residency**	
Yes	16 (25.4)
No	4 (6.3)
Missing	43 (68.3)

**Figure 5.**
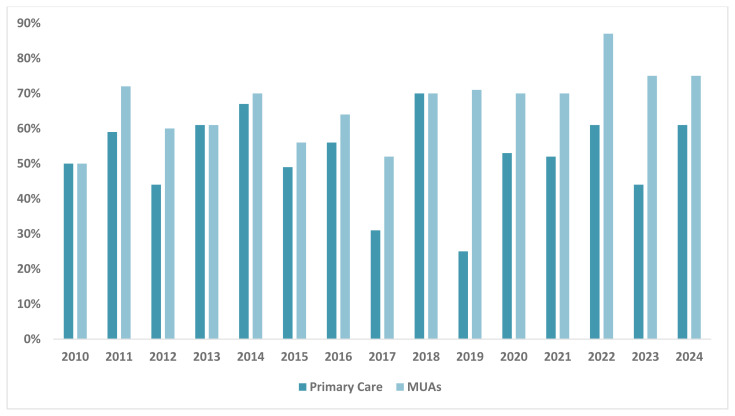
Percentage of PCM Graduates Who Went into Primary Care Practice and in Medically Underserved Areas, 2010 – 2024, N = 395. This graph illustrates the distribution PCM graduates who entered primary care practice and those who went on to serve in medically underserved areas. Graduates pursuing primary care were categorized into Internal Medicine, Family Medicine, and Pediatrics. Medically underserved areas are defined as regions with shortage of primary care health services.

***Program impact on career choice.*** A third of the respondents reported that the PCM Program impacted their specialty selection, with an additional quarter acknowledging its influence on their residency choice ( Table 1). Of the PCM graduates, 53% pursued careers in primary care, while 67% opted to practice in MUAs ( Figure 5).

***Program impacts on ability to practice patient-centered care.*** Of the respondents who completed the questions regarding the program preparing them for their medical career, 60% reported that the program prepared them to practice patient-centered care for all patients, and 85% reported that the program prepared them to provide patient-centered care for underserved and vulnerable patients ( Table 2).

**Table 2.** PCM Program Preparation, n = 48.

Preparation to practice patient-centered care *for all patients*	Frequency (%)
A great deal	29 (60.4)
Somewhat	19 (39.6)
Preparation to practice patient-centered care *for underserved and vulnerable patients*	
A great deal	41 (85.4)
Somewhat	7 (14.6)

The most important PCM skills reported by respondents that they applied in practice included: building relationships with patients (56%); communicating well with patients (56%); and practicing collaborative decision-making with patients (48%) ( Table 3).

**Table 3.** Most Important Skills Applied in Practice from PCM Scholars Program, N = 63.

Skills Applied in Practice	Frequency (%)
Building relationships with my patients	35 (55.6)
Communicating well with my patients	35 (55.6)
Practicing collaborative decision making with my patients	30 (47.6)
Facilitating coordination and integration of care for my patients	24 (38.1)
Educating my patients	16 (25.4)
Other	4 (6.3)

Of the respondents who completed the question regarding PCM Behaviors that the program prepared them to apply in practice, 93% selected almost always working to understand their patient by discussing their beliefs about their medical conditions; 100% selected almost always reviewing options with the patient regarding the next steps of the workup and management in order to make their patient an integral part of the decision-making process; 95% selected almost always effectively engaging other health care professionals ( Table 4).

**Table 4.** Respondents who ‘Almost Always’ Practice Patient Centered Medicine Behaviors, n = 40.

PCM Behaviors	Frequency (%)
	Almost Always
**Working to understand the patient’s ideas, feelings, worries, and concerns by eliciting and discussing the patient’s**	
Most pressing or important concerns	37 (92.5)
Beliefs about their medical condition(s)	28 (70.0)
Psychosocial stressors or non-medical factors that may impact their mental and/or physical health	37 (92.5)
Concerns regarding navigating the medical system	28 (70.0)
**Making patients an integral part of the collaborative decision-making process by**	
Discussing / sharing what I think about the problem by fully informing my patients about their medical situation and exploring the patient’s perspective to reach mutual agreement on treatment plans	39 (97.5)
Reviewing options with the patient regarding next steps of the workup and management	40 (100.0)
Eliciting the patient’s cultural beliefs and practices, as well as socioeconomic factors that need to be considered in the care plan	27 (67.5)
Respecting my patients’ views even though I may not be able to incorporate them into their care plan	40 (100.0)
Seeking the patient’s input about the feasibility of the management plan including discussion of possible barriers that might interfere with adhering to the plan	36 (90.0)
Incorporating patient empowerment strategies, such as motivational interviewing and action-planning, to increase the likelihood of adherence to the care plan	21 (52.5)
Explicitly confirming at the end of the encounter the patient’s understanding of the mutually agreed upon next steps	35 (87.5)
**Facilitate continuous, coordinated, and comprehensive care for patients**	
Advocate for my patients’ rights	32 (80.0)
Help my patients navigate the health care system	40 (100.0)
Facilitate my patients’ use of community resources in the management of their health issues	25 (62.5)
Help my underserved patients access care	34 (85.0)
Effectively engage other health care professionals, e.g., nurses, social workers, nutritionists, pharmacists, in the care of my patients.	38 (95.0)
Help my patients develop strategies to overcome difficulties with a complex chronic disease management problem.	32 (80.0)

### Qualitative analysis

***Exposure to patient experience.*** In analyzing the comments from the open-ended question regarding what the respondents found to be most valuable in the PCM Scholars Program, one major theme emerged: *Exposure to Patient Experience*. Program graduates reported that their interactions with patients added great value to their medical education.

 The following quotes illustrate what respondents found to be valuable in the program:

*“Face-to-face interactions with patients in the clinical setting put me ahead of the curve throughout medical school and in residency. I was able to be aware of all the necessary components of providing comprehensive care.”*
*“It was an eye-opening experience to work with an HIV free housing community. Something not taught in traditional medical school curriculum.”*

***Dedication to serving the underserved and building relationships with patients.*** In the open-ended question regarding how participating in the program influenced respondents’ work after graduation, two major themes emerged: 1. *dedication to serving the underserved*, and 2. *building relationships with patients*. Respondents reported that they were able to learn to be better physicians, empathize, and to better communicate with patients.

 The following quotes highlight how the program influenced their work after graduation:

*“It helped me to understand different patient perspectives and empathize with my patients better. Thus, translating into better patient care.”*
*“Getting to work with HIV patients and understanding their unique need for access to non-medical resources as a means to improve their health helped me appreciate barriers that exist for so many underserved communities.”*

***Shared decision making, identifying and adapting to patients’ needs and compassionate care.*** Three themes emerged in response to how respondents defined patient-centered care: *shared decision-making*, *identifying*, and *adapting to patients’ needs and compassionate care*. When approaching patient-centered care, respondents reported applying a holistic approach to providing care to their patients.

 The following quotes demonstrate how respondents’ approach and define patient-centered care:

*“Shared decision-making to help patients achieve healthcare goals.”*
*“Care that recognizes the patient's role in their health care, including attitudes, prejudices, feelings, priorities.”*
*“Taking into account not only the medical issues, but also the social aspects of a person and trying to treat the whole person and sometimes even including the family.”*

***Other aspects of professional development, work, motivation, or aspirations that were influenced by the PCM Scholars Program.*** Three main themes emerged in response to the open-ended question about other aspects of respondents’ professional development, work motivation, or aspirations that were influenced by the program: *the value of the faculty mentors*, *the integration of medicine and public health*, and *the motivation to practice patient-centered medicine.*

Illustrative quotes from respondents regarding the value of the faculty mentors:

*“Opportunity to meet and learn from such a passionate group of mentors and peers. Be inspired by their commitment and advocacy”*
*“I think my mentors modeled well how to be a mentor for others and create an open and collaborative environment”.*
*“The continuity of instruction and relationships I developed with the advisors/teachers/mentors in PCMS was invaluable to calming my nerves when I changed what I was doing at least once every 3 months”*
*“The love from Dr Hasnain is the secret ingredient!”*

Illustrative quotes from respondents regarding the integration of medicine and public health:

*“It was an introduction to the overlap between public health and clinical medicine, demonstrating the importance of practicing at the interface between these two complementary fields.”*
*“It was an introduction to the overlap between public health and clinical medicine, demonstrating the importance of practicing at the interface between these two complementary fields.”*

Illustrative quotes from respondents regarding the motivation to practice patient-centered medicine:

*“I think I still see that time as an ideal level of understanding my patients' experiences/values/beliefs, and I constantly strive to achieve some small percentage of that as I encounter my new patients.”*
*“Keeping my interest in medicine alive.”*
*“I considered it a defining aspect of my training at UIC and the type of training that UIC is particularly skilled at providing. My training in PCM helped me set vulnerable populations as a priority in my practice and research and, more importantly, set community organizations in general as essential partners in addressing big picture issues such as disparities in care.*

## Discussion

 One of the greatest concerns consistently voiced by national agencies, institutions, educators, and researchers is that physicians undervalue the experiences and perspectives of patients regarding their illness and its management. Contributing to this problem is the growing complexity and fragmentation of the healthcare system. This results in clinicians feeling more pressured to see more patients in less time, thus impacting continuity of care, a central feature of an effective patient-physician relationship. Care has become increasingly centered not on patients' needs but on the system's needs.

The current structure of medical school education does not enable students to learn to provide continuity of care; clinical and academic experiences are geared toward episodic learning. Not surprisingly, the literature confirms that training in patient-centered care in undergraduate medical education is inadequate in its current state. The infrequent training that does take place occurs in the clinical and residency years; it typically does not take place in the pre-clinical years, although some programs have introduced courses or learning activities to teach elements of patient-centered care and health equity ^10– 13^. For example, at Wake Forest School of Medicine, a longitudinal cohort study was conducted to evaluate the association of a health equity curriculum with medical students’ self-reported outcomes ^39^. Significant improvements were found in student’s self-reported knowledge of social determinants of health and confidence in working with underserved populations.

Our study sheds light on the impacts of training in patient-centered medicine on graduates’ career choices and practice behaviors, and the findings are encouraging. One of the important issues in addressing the health needs of our growing and evolving patient populations is to have an adequate physician workforce that is able and committed to careers in primary care and serving the underserved ^40^. Our training program seems to impact graduates pursuing primary care as the data demonstrates that more than half of PCM graduates decided to go into primary care, and more importantly, 67% chose medically underserved areas for residency training.

 At the time of the design of the PCM Scholars Program, the longitudinal primary care (LPC) program at the University of Illinois Chicago was time and task-oriented rather than patient-oriented. In the M1 and M2 years, students saw their LPC preceptor only sporadically and primarily functioned as observers rather than as active participants in taking care of patients. Further, students rarely saw the same patient more than once. Classroom sessions in the pre-clinical years tended to be topic-based and rarely helped students make connections to real patients. Clerkship clinical experiences had limitations as well; though students spent six weeks at one clinical site, data indicated that similar to their LPC experiences, they seldom see the same patient more than once. The fourth-year experience was characterized by specialty rotations and electives, emphasizing specific knowledge and skills. All the above factors, which are common to many medical schools, work against students’ learning the true meaning of patient-centered care through patient experiences grounded in continuity of care.

 The PCM Scholars Program positively impacts students' choice of primary care and their ability to work in medically unserved areas. In addition, the findings indicate that the program prepared the graduates to practice patient-centered care for all patients, particularly for underserved and vulnerable populations. According to the qualitative analysis, respondents reported that the program helped solidify and influence their choice of working in primary care and serving the underserved. The following are selected quotes from scholars who emphasized how pivotal the program was in their medical careers:

*“I considered it a defining aspect of my training at UIC and the type of training that UIC is particularly skilled at providing. My training in PCM helped me set vulnerable populations as a priority in my practice and research and, more importantly, set community organizations in general as essential partners in addressing big picture issues such as disparities in care.”*
*“This program was by far the most influential part of my medical school training.”*

It is important to acknowledge the limitations of the study. First, the study's cross-sectional design only provides information from a single moment in time, and future research utilizing a longitudinal design is warranted to further demonstrate the impacts of the PCM Scholars Program on graduates’ career choices and practice behaviors. Second, graduates' response rate was low (33%) when the survey was disseminated even after three follow-ups. Further, other factors may influence graduates’ career choices; evidence shows that those who attend a public institution tend to venture into primary care ^41, 42^.

## Conclusions

 This paper presents findings of an evaluation of the impacts of the Patient-centered Medicine Scholars Program, developed and implemented at a public medical school, the University of Illinois College of Medicine, at its Chicago campus. The findings provide valuable information about how training in patient-centered medicine impacted program participants regarding their post-graduation career choices and practice behaviors. Qualitative findings also highlighted the PCM Scholars Program’s influence on trainees' professional development via the value of the faculty mentors, the integration of medicine and public health, and the motivation to practice patient-centered medicine. The PCM Scholars Program contributes to our institutional values and goals and is transformative in educating students to become patient-centered physicians. Addressing the persisting healthcare disparities and advancing health equity requires not only redesigning training programs to better prepare the future health workforce but also sharing the work we do, so that we can benefit from best practices and lessons learned ^41^. This information may also guide and inform other institutions about ways to incorporate longitudinal patient-centered care training into health professions education to optimize the preparation of a 21 ^st^-century health workforce that would meet the needs of diverse patients and communities and advance health equity.

## Ethics and consent statement

The study was reviewed and approved by the University of Illinois Chicago (UIC) Institutional Review Board (IRB) [IRB Protocol Number: 2007-0567] as exempt on June 19, 2017. All study procedures were conducted in accordance with the ethical standards of the UIC IRB and with the principles outlined in the Declaration of Helsinki (2013) for research involving human subjects. The IRB approved a waiver of documented written consent due to the minimal risk nature of the study and low participant burden. Consent was implied through voluntary completion of the survey, as approved by the IRB. Participation was voluntary, and participants were assured of the confidentiality and anonymity of their responses.

## Data Availability

Access to the dataset is restricted in accordance with guidance from the UIC IRB. Although the dataset does not include identifiable or sensitive information, the IRB-approved protocol limits sharing due to ethical considerations related to participant consent and data governance. Researchers who wish to access a de-identified or limited version of the dataset may submit a formal request to the corresponding author. Requests must include a summary of the proposed research and how the data will be used.
