# Supplementary material for: Impacts of longitudinal training in patient-centered medicine on trainee physicians’ career choices and practice behaviors
Source: MedEdPublish (2016). 2025 Sep 18;15:130. [Version 1] doi: 10.12688/mep.20993.1 (PMC12780525; doi:10.12688/mep.20993.1)
Supplement: Peer review history

# Reviewer report: Reviewer response for version 1

**DOI:** 10.21956/mep.22486.r45042

**Published:** 2026-01-23

**Authors:** Anna Chur-Hansen

**Affiliations:** 1 School of Psychology, Faculty of Health and Medical Sciences, University of Adelaide, Adelaide, SA, Australia

Thank you for the opportunity to read about your longitudinal curriculum design and feedback from trainees regarding its impact on their career choices and self reported practice behaviours.

 My main feedback pertains to the qualitative arm of your study.  You indicate that you have used Content Analysis (CA).  Typically, in CA, the qualitative data would be reduced to counts of codes, with illustrative quotes for codes.  I see that the reference you draw upon for the method is from 1989.  There are numerous more recent methodology papers on CA and what I think is more relevant for your paper, Qualitative CA – see for example

 Hsieh HF, Shannon SE. Three approaches to qualitative content analysis. Qual Health Res. 2005 Nov;15(9):1277-88. doi: 10.1177/1049732305276687. PMID: 16204405.

 For replication you need to explain how qualitative data were (a) collated for analysis; (b) coded (including by whom); (c) provide either IRR or trustworthiness evidence, for rigour; (d) usually as I have said above, for CA there would be counts – so categories and illustrative quotes.  These would be in a table.

 For QCA you may have themes – but not CA.  Again, you can have illustrative quotes – but these should not be listed one after the other – there would be one illustrative quote that you feel best exemplifies the code or category.

 There should never be new findings/results (including quotes from qualitative data) in the Discussion.

 I hope this is useful advice – qualitative data needs to be as carefully and as transparently analysed as quantitative data are.

Have any limitations of the research been acknowledged?

Partly

Is the work clearly and accurately presented and does it cite the current literature?

Yes

If applicable, is the statistical analysis and its interpretation appropriate?

Yes

Are all the source data underlying the results available to ensure full reproducibility?

Partly

Is the study design appropriate and is the work technically sound?

Yes

Are the conclusions drawn adequately supported by the results?

Partly

Are sufficient details of methods and analysis provided to allow replication by others?

No

Reviewer Expertise:

NA

I confirm that I have read this submission and believe that I have an appropriate level of expertise to confirm that it is of an acceptable scientific standard, however I have significant reservations, as outlined above.

# Reviewer report: Reviewer response for version 1

**DOI:** 10.21956/mep.22486.r45044

**Published:** 2026-01-07

**Authors:** Sunny Nakae

**Affiliations:** 1 California University of Science and Medicine School of Medicine, Colton, CA, USA

This study adds to extant literature in medical education that experiences and exposure during medical school can shape career and practice decisions for trainees. The core skills and components of patient centered medicine can be effectively taught within a curricular framework that is delivered longitudinally, and these core skills have a general benefit while also encouraging trainees to pursue primary care fields. The focus on vulnerable populations and psychosocial/systemic factors is particularly important, as it highlights the importance of structural mechanisms that influence health outcomes and patient behaviors. 

 The unique element of longitudinal care highlights how this change in design is critical in helping trainees build skills in relationships, communication, trust, and systems awareness.  The intermittent model of preceptorships in contrast to the long term care patient home is a critical contribution to medical education. 

 A curiosity provoked by this article is whether the attention to social determinants of health within UME curricula has increased since the initial scoping study in 2020 and 2019.  I also wonder the impact on individuals that pursued non-primary care areas and what their assessment of the program on their careers is. 

 The response rate for graduates in the cross-sectional sample was low, which should be mentioned as a limitation.  This may indicate some favorability bias that those who were committed to underserved communities or in  primary care fields were more motivated to respond. 

 In summary this article confirms that core skills, values, and patient centered medicine practices can be effectively taught in UME and that there is evidence that these skills can translate into career decisions and self-reported practice behaviors in physicians.

Have any limitations of the research been acknowledged?

Yes

Is the work clearly and accurately presented and does it cite the current literature?

Yes

If applicable, is the statistical analysis and its interpretation appropriate?

Yes

Are all the source data underlying the results available to ensure full reproducibility?

Yes

Is the study design appropriate and is the work technically sound?

Yes

Are the conclusions drawn adequately supported by the results?

Yes

Are sufficient details of methods and analysis provided to allow replication by others?

Yes

Reviewer Expertise:

Medical education pathways, underserved communities, mentorship, structural vulnerability, structural competency, bias mitigation, enrollment management, educational disparities and access.

I confirm that I have read this submission and believe that I have an appropriate level of expertise to confirm that it is of an acceptable scientific standard.

# Reviewer report: Reviewer response for version 1

**DOI:** 10.21956/mep.22486.r43598

**Published:** 2025-10-31

**Authors:** Ingrid Philibert

**Affiliations:** 1 School of Medicine at Quinnipiac University, North Haven, USA

Strengths include meaningful outcomes: 53% of graduates chose primary care, and 67% work in medically underserved areas. Graduates report strong preparation for patient-centered care, especially for vulnerable populations. The emphasis on social determinants and health disparities is well-aligned with some calls for reform in medical education.

 Aspects that could be strengthened include addressing missing data or unclear formatting in some tables (Table 1). The cross-sectional design of the study captures only a snapshot in time. This should be noted in the limitations and note that future research should consider longitudinal tracking of graduates to assess sustained impact. The survey response rate was 33%, which may limit generalizability. Clarify how missing data were handled and any implications for interpretation.

 The limitations section could be strengthened by discussing other confounding factors (e.g., institutional culture, external influences) that may affect career choices. The program is described in the context of a single institution. This section also should discuss how findings may or may not translate to other settings or institutions with different populations and resources.

Have any limitations of the research been acknowledged?

Yes

Is the work clearly and accurately presented and does it cite the current literature?

Yes

If applicable, is the statistical analysis and its interpretation appropriate?

Yes

Are all the source data underlying the results available to ensure full reproducibility?

Yes

Is the study design appropriate and is the work technically sound?

Yes

Are the conclusions drawn adequately supported by the results?

Yes

Are sufficient details of methods and analysis provided to allow replication by others?

Yes

Reviewer Expertise:

Medical Education, with a focus on the transition from undergraduate to graduate medical education.

I confirm that I have read this submission and believe that I have an appropriate level of expertise to confirm that it is of an acceptable scientific standard.
